# Diagnostic value of OSA-18 scale combined with the lowest blood oxygen saturation at night in children with obstructive sleep apnea

**DOI:** 10.3389/fped.2026.1734784

**Published:** 2026-01-23

**Authors:** Peiyuan Su, Yanling Yuan, Huiling Liao, Yinghong Fan, Tao Ai

**Affiliations:** Department of Pediatric Pulmonology, Chengdu Women's and Children's Central Hospital, School of Medicine, University of Electronic Science and Technology of China, Chengdu, China

**Keywords:** children, obstructive sleep apnea, OSA-18 scale, oxygen saturation, polysomnography

## Abstract

**Background:**

The incidence of obstructive sleep apnea (OSA) in children has increased in recent years. Many primary hospitals have not polysomnography which can diagnose OSA. To investigate the lowest blood oxygen saturation at night (LSaO_2_) combine with obstructive sleep apnea 18 items survey (OSA-18) scale to initially screen the sleep status of children with OSA.

**Methods:**

A retrospective study of 189 children aged 4–12 years with sleep-disordered breathing was conducted. All children were monitored using polysomnography and divided into the simple snoring and OSA groups (mild, moderate, and severe). Their parents completed the OSA-18 scale. Correlation among three indices [LSaO2, OSA-18 scale, and obstructive apnea hypoventilation index (OAHI)] was assessed. Subsequently, series and parallel tests were used to understand the sensitivity and specificity of diagnosis.

**Results:**

There was no statistical differences in sex (*P* = 0.909) and age (*P* = 0.894), and a significant difference in OSA-18 scores between the simple snoring and OSA groups (*P* = 0.014) but not in the LSaO2 (*P* = 0.409). OSA-18 and LSaO2 scores of the mild, moderate, and severe groups were significantly different (*P* < 0.05), and LSaO2 was correlated with the OAHI. Use of the OSA-18 scale combined with LSaO2 to assess the boundary value of mild and moderate-to-severe OSA was better than that of each index independently. The sensitivity and specificity the parallel test were 85.7% and 62.7%, respectively, can better predict OSA than series test.

**Conclusion:**

The OSA-18 scale combined with LSaO2 has diagnostic value for the diagnosis of OSA, and can be used as a prediction tool for OSA.

## 1 Introduction

In recent years, the incidence of sleep-disordered breathing in children has steadily increased, with an incidence rate of 10.8% (1). Sleep-disordered breathing in children includes obstructive sleep apnea (OSA), central sleep apnea, and hypopnea, among which OSA is the most common. Occurring in children aged 2–8 years, it is characterized by partial upper airway obstruction (obstructive hypopnea) and/or intermittent complete obstruction (obstructive apnea), disturbance of ventilation during sleep, and sleep pattern disorders (2). The prevalence of OSA in children in China is also relatively high, with a reported prevalence of 4.8% in Hong Kong and China in 2010 (3). At present, the gold standard for diagnosing OSA is polysomnography (PSG), the “Guidelines for the Diagnosis and Treatment of Obstructive Sleep Apnea in Chinese Children (2020)” (hereinafter referred to as the “Guidelines”) pointed out the OSA severity grading criteria for children: 1 < OAHI ≤ 5, 5 < OAH I ≤ 10, and OAHI > 10, is mild, moderate and severe, respectively. But this procedure requires an overnight stay in a special sleep monitoring room. It is a complicated and expensive test, time-consuming for the parents of the child. It requires high technical level of the operator. Thus, it is not carried out in many primary hospitals. The OSA-specific quality of life for children with obstructive sleep apnea 18 items survey (OSA-18) scale is one of the most widely used and specific scales to investigate the quality of life of children with OSA at the world (4, 5). It can effectively reflect the quality of life of children and can predict the degree of disease and treatment effect in children with OSA to a certain extent. However, the “Guidelines” 2020 Edition pointed out that the diagnostic accuracy of questionnaires alone is not high, and so they need to be considered in combination with medical history, physical examination, and sleep monitoring equipment (2). The lowest blood oxygen saturation at night (LSaO_2_) is an important indicator of sleep disorder severity, The examination can be completed with blood oxygen monitor, which is easy to perform. The purpose of this study was to combine the LSaO_2_ and OSA-18 scales to initially screen the sleep status of children with sleep-disordered breathing and study the feasibility of predicting OSA.

## 2 Materials and methods

### 2.1 Research participants

Among preschool children, adenoid hypertrophy is the main cause of OSA in preschool children of normal weight. The adenoid reaches its peak of physiological hyperplasia from 2 to 6 years old. After about 12 years old, the volume of the adenoid significantly shrinks. Because it is difficult for children under 4 years old to complete polysomnography, so 4- to 12-year-old children with sleep-disordered breathing who were treated in the Pediatric Respiratory Department of Chengdu Women's and Children's Central Hospital from August 2023 to October 2024 were selected. The inclusion criteria were as follows: 1) children with nocturnal sleep snoring, Mouth breathing, apnea, frequent awakening, and other symptoms over a duration of ≥3 months.2) children and their parents showing good compliance and ability to complete the examination and the questionnaire. The exclusion criteria were as follows: parents of children with congenital upper airway dysplasia, symptoms of upper respiratory tract infection when they came to the hospital, parents of children who refused to answer the questionnaire, and children with poor compliance.

### 2.2 Research methods

#### 2.2.1 PSG

The enrolled children were monitored by PSG using a Philips Alice 6 PSG instrument, and they were divided into the simple snoring group (OAHI ≤ 1) and OSA group (OAHI > 1) according to the results (1). The OSA group was further divided into the mild group (1 < OAHI ≤ 5), moderate group (5 < OAH I ≤ 10), and severe group (OAHI > 10). (Unit of OAHI: event/h).

#### 2.2.2 OSA-18 scale

The staff helped the parents of the enrolled children to complete the OSA-18 scale. After explaining the requirements to the parents, they filled out the form immediately and submitted it to professionals for collection and review. If there were omissions or unclear content, it was immediately improved and corrected.

Since parents fill out the questionnaires with the assistance of staff, there may be some subjective human factors that affect the objectivity of the results. Before the research was launched, we provided standardized training to the staff and gave a standard explanation of the scale content. Moreover, they only offered explanations and assistance when parents raised questions, avoiding the use of guiding language, and did not participate in parents' filling at other times. This can ensure the standardized operation of the questionnaire to the greatest extent.

The OSA-18 scale includes five dimensions: sleep disturbance, physical symptoms, poor mood, daytime functional status, and degree of influence on the child's guardian. Each dimension is further divided into three and four items. The frequency of symptoms was recorded on a 7-point scale: 1 = none, 2 = almost never (0–1 time/month), 3 = very rarely (2–3 times/month), 4 = sometimes (1–2 times/week), 5 = often (3 times/week), 6 = mostly (once every other day), and 7 = every day. The total score ranges from 18 to 126, with higher scores indicating a more severe impact on the quality of life. A score of <60 indicates mild, 60–80 indicates moderate, and >80 indicates severe.

### 2.3 Statistical analysis

After the data of the two groups of children were collected, SPSS version 26.0 (IBM, Armonk, NY, USA) was used for analysis, and the enumeration data were analyzed using the χ^2^ test. The data were subjected to the Kolmogorov–Smirnov test. Normally distributed data are expressed as the mean ± standard deviation ($\bar{\chi }\;\;\pm \;S$), and the independent samples *t*-test was used. Non-normally distributed data were subjected to the nonparametric test. Pearson's test was used to analyze the correlation between each index of the OSA-18 scale and the OAHI and the receiver operating characteristic (ROC) curve was used to conduct diagnostic tests of LSaO_2_ combined with the OSA-18 scale and determine the optimal critical value. The diagnostic tests were compared using four fold table. *P* < 0.05 indicated that the difference was statistically significant.

## 3 Results

### 3.1 Research participants

A total of 189 patients were selected and divided into simple snoring and OSA groups according to the OAHI. The amount of simple snoring group is 15, the amount of OSA group is 174.Data for each group are presented in Table 1. Children diagnosed with OSA were divided into three groups: mild, moderate, and severe. Data for each group are presented in Table 2.

**Table 1:** Baseline characteristics of children in each group.

Index	Number *n* = 189	Sex (male/female)	Age (years)	OAHI	OSA-18 score	LSaO_2_
Simple noring group	15	9/6	5.8 ± 1.7	0.8 ± 0.1	47.9 ± 9.3	90.3 ± 6.4
OSA group	174	107/67	6.0 ± 1.9	6.2 ± 7.6	56.2 ± 16.8	88.3 ± 6.1
χ^2^**/***Z*		0.013	−0.133	6.421	−2.678	−2.15
*P-*value		0.909	0.894	0.000	0.014	0.409

**Table 2:** Comparison of OSA-18 scores and LSaO_2_ between OSA children in each group.

Index	Number *n* = 174	Sex (M/F)	Age (years)	OAHI	OSA-18 scores	LSaO_2_
Mild	124	77/47	6.0 ± 1.9	2.9 ± 1.3	54.3 ± 15.7	89.2 ± 6.2
Moderate	28	16/12	5.9 ± 0.2	8.2 ± 3.0	55.6 ± 16.8	88.1 ± 4.0
Severe	22	14/8	6.0 ± 1.6	22.8 ± 10.0	67.5 ± 19.1	83.4 ± 6.1
χ^2^/*Z*/*t*		0.286	0.382		−0.152^a^	1.708
					−3.517^b^	−2.723
					2.424^c^	4.898
*P-*value		0.867	0.826		0.879^a^	0.263^a^
					0.001^b^	0.019^b^
					0.015^c^	<0.0005^c^

^a^ Mild vs. moderate.

^b^ Mild vs. severe.

^c^ Moderate vs. severe.

### 3.2 Correlation analysis of LSaO_2_, OSA-18 scores, and OAHI

**Figure 1:**
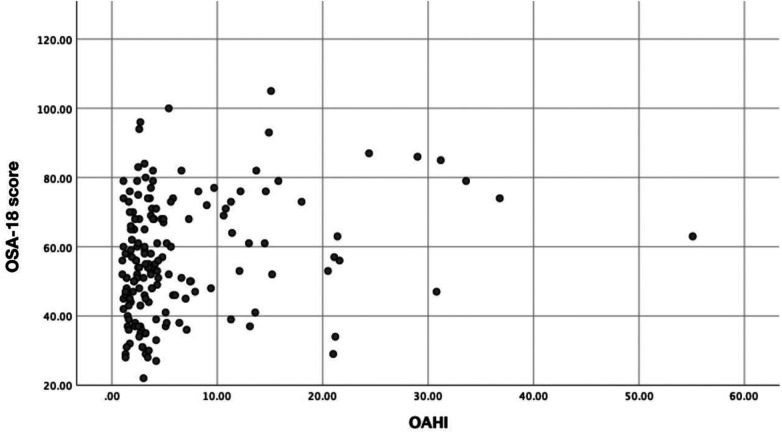
Scatter plot of the correlation between OSA score and OAHI in OSA group.

**Figure 2:**
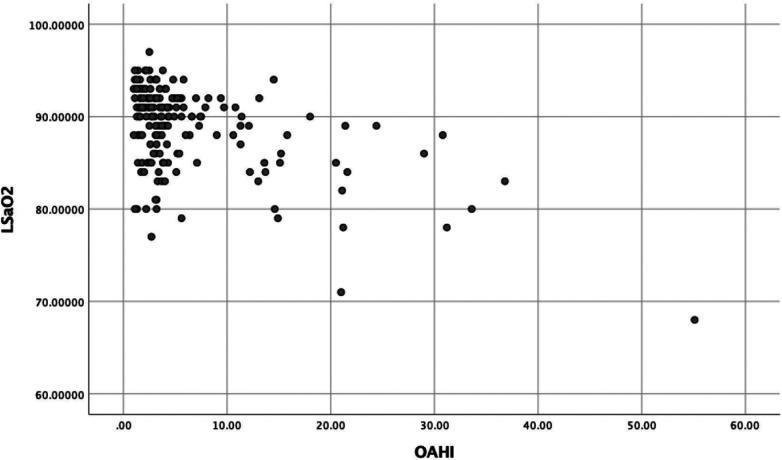
Scatter plot of the correlation between OAHI and LSaO_2_ in OSA group.

In the OSA group, all dimensions of the OSA-18 score, except daytime function and emotional symptoms, were correlated with the OAHI (*P* < 0.05). Data for each group are presented in Table 3. Figure 1 show the positive correlation between the OSA-18 scores and OAHI, and Figure 2 show the negative correlation between LSaO_2_ and OAHI.

**Table 3:** Analysis of the correlation between OSA-18 scores, LSaO_2_, and OAHI.

Index	OSA-18 score							LSaO_2_
	Domain	Sleep disturbance	Physical symptoms	Emotional symptoms	Daytime functions	Care giver concerns	Total	
	Score	9.1 ± 3.3	13.6 ± 4.2	9.1 ± 4.3	8.3 ± 4.3	16.0 ± 7.4	56.2 ± 16.8	
Correlation with OAHI	*r*	0.261	0.155	0.082	0.1	0.252	0.246	−0.355
	*P*-value	<0.001	0.041	0.285	0.189	0.001	0.001	<0.001

### 3.3 ROC curve analysis results of the OSA-18 scale combined with LSaO_2_ in the diagnosis of mild and moderate-to-severe OSA

**Figure 3:**
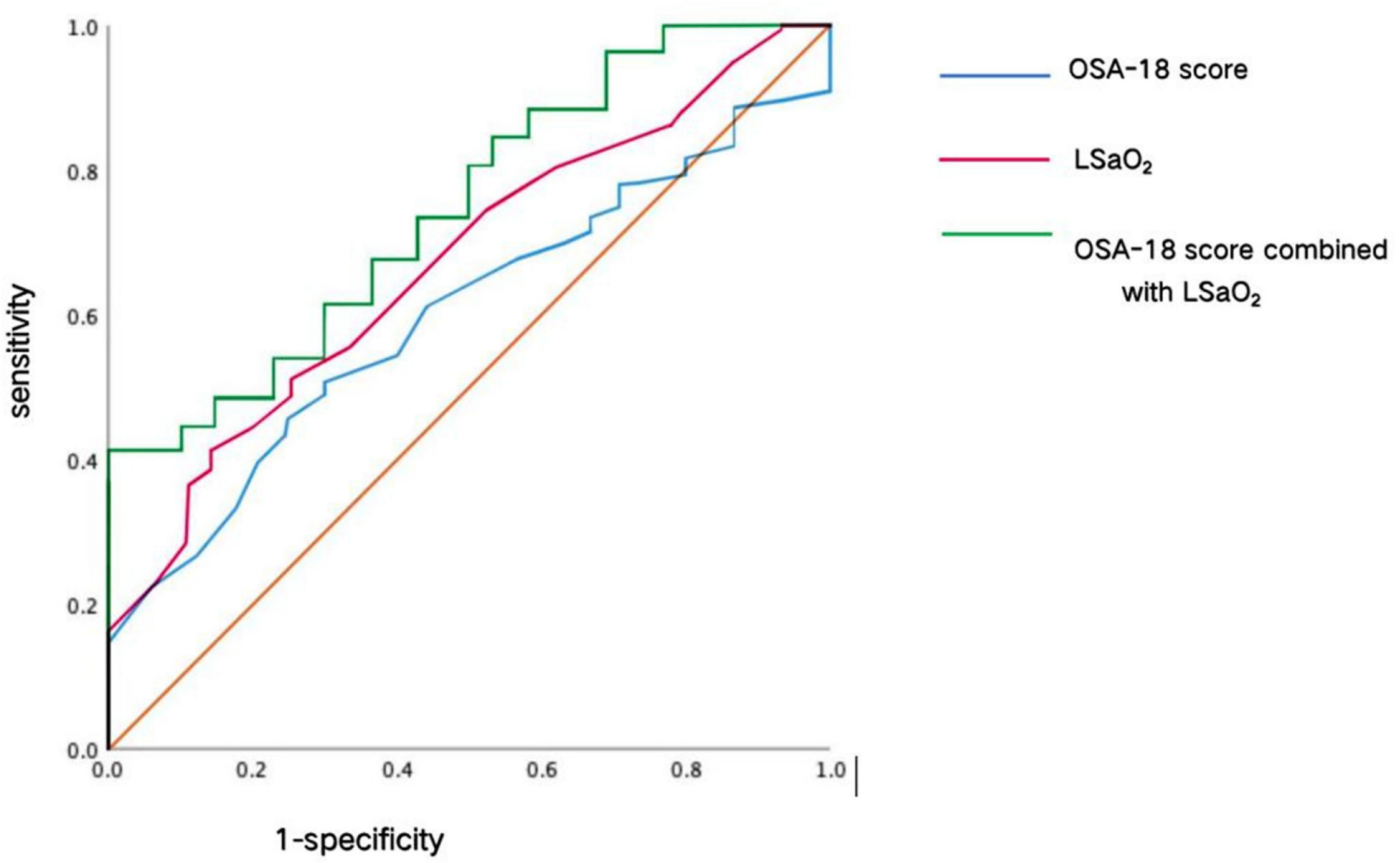
Results of ROC curve.

Based on the OAHI to judge OSA severity, children in the OSA group were divided into mild and moderate-to-severe OSA groups, and the total scores of OSA-18 and LSaO_2_ were used as indicators. The ROC curve was then drawn to understand the total score of OSA-18 combined with LSaO_2_ to determine the OSA diagnostic index. The results showed that the area under the curve (AUC) of the OSA-18 total score combined with LSaO_2_ was larger than that of the OSA-18 total score and LSaO_2_ alone (AUC 0.739), and the specificity was also higher than that of the two separate datasets (0.821). However, the sensitivity decreased (0.466). The results are presented in Table 4 and Figure 3.

**Table 4:** Results of ROC curve analysis.

Index	AUC	*P*	Youden index	Best cut-off	Specificity	Sensitivity	95% CI
OSA-18 score	0.623	0.009	0.223	59.5	0.669	0.554	53.3–71.4
LSaO_2_	0.669	<0.001	0.258	90.5	0.75	0.517	58.6–75.2
OSA-18 score combined with LSaO_2_	0.739	<0.001	0.287	–	0.821	0.466	66.4–81.5

### 3.4 Specific test analysis of the OSA-18 total score combined with LSaO_2_ in the diagnosis of mild and moderate-to-severe OSA

The total OSA-18 and LSaO_2_ scores were tested in series and in parallel, and the diagnostic standard was the best critical value on the ROC curve. The total score of the OSA-18 was 59.5, and the LSaO_2_ was 90.5%. The sensitivity of the parallel test was 85.7%, which was higher than that of the series test. Although the sensitivity was high, the specificity decreased to 62.7%. The results are presented in Table 5.

**Table 5:** Diagnostic test of LSaO_2_ combined with the OSA-18 total score for OSA in children.

Index	Diagnostic test	Sensitivity % [95% CI (%)]	Specificity% [95% CI (%)]
OSA-18 fraction + LSaO_2_	Tandem test	44.6 (31.5–58.4)	81.3 (72.9–87.7)
	Parallel test	85.7 (73.2–93.2)	62.7 (53.2–71.2)

## 4 Discussion

Upper airway obstruction is the pathological cause of OSA, which include morning headache, drowsiness, nasal congestion, and mouth breathing, adenoid appearance in severe cases, abnormal behavior, irritability, lethargy, inattention, and recurrent upper respiratory tract infection. Children with long-term symptoms may experience growth retardation, hypertension, cardiac enlargement, right heart failure, and pulmonary heart disease. The early symptoms include snoring, mouth breathing, daytime sleepiness, and decreased concentration. Early identification and intervention can reduce the long-term harm caused by OSA (6). At present, the gold standard for diagnosing this disease is PSG, but because of its high requirements on the site, in terms of equipment, inspection time, and professional level of operators, and due to the age of children, the process of installation, unfortunately, PSG is a diagnostic instrument that cannot be matched, resulting in a failure of detection. Therefore, many primary hospitals fail to conduct such inspections. With the deepening of doctors' and parents' understanding of OSA, many people have realized the long-term harm of OSA to children, and an increasing number of children come to the hospital for sleep-disordered breathing. Primary physicians urgently need a simple and effective diagnostic tool for the initial screening and identification of OSA.

The OSA-18 scale, was developed by Franco in 1999 (4). It is the most commonly used OSA quality of life evaluation scale for children that reflects the children's quality of life and physical and mental health by recording the subjective feelings of the children and their parents. A total of 60 and 80 points are used to score the severity of the affected quality of life. Its Cronbach's coefficient is 0.65 (7). However, the Guidelines pointed out that the accuracy of using the scale alone to diagnose OSA is not high, and it cannot be used as a substitute for PSG. Therefore, it must be combined with other clinical diagnostic tools (1). Our research found a correlation between OAHI and the total score of OSA-18. Among the five indicators of the OSA-18 scale, OAHI is correlated with three indicators: sleep disturbance, physical symptoms, and Care giver concerns, but no correlation with poor mood and daytime function.

We believe that when children show symptoms of OSA, their sleep disturbance such as “snoring”, “frequent awakens”, “breathing pauses” and other symptoms, as well as physical symptoms like “increased nasal discharge” and “frequent colds”, are likely to draw the attention of parents. However, “poor mood” and “hyperactive behaviors”, as well as “frequent naps during the day” and “inattentiveness”, are often overlooked by parents. The reason for the analysis may be that parents generally pay more attention to physical pathological symptoms but tend to ignore their children's emotional and psychological states. Second, parents tend to work during the day and have less contact with their children in the day than when they go home at night; thus, the questionnaire data in this regard may be less accurate, so parents often fail to notice their children's abnormal functions during the daytime.

Domestic research in this area is also controversial, and there are differences in the correlations between several dimensions of the scale and OAHI. The Guidelines also suggest that this scale needs to be comprehensively evaluated in combination with the results of instrument testing and to evaluate OSA from both subjective and objective perspectives. The results of this study suggest that the OSA-18 score is 59.5, which is the cut-off value for mild and moderately severe OSA and is basically the same as the OSA-18 scale. The guideline points out that tonsillectomy and/or adenoidectomy is currently one of the first-line treatment methods for children with OSA, especially for children with moderate to severe OSA. After a comprehensive assessment of the upper airway condition (including the nose, nasopharynx, oropharynx, laryngopharynx and larynx) through endoscopy or imaging, and when the clinical examination shows that the tonsils and/or adenoids are enlarged and there are no surgical contraindications, it is his preferred treatment method.

LSaO_2_ can be monitored by PSG, and blood oxygen monitor can be used if PSG is not available. The 2020 edition of the Guidelines does not include LSaO_2_ in the diagnostic criteria for OSA, and it is believed that the specificity and sensitivity of this indicator need further research (2). The McGill Oximetry Score (MOS) is recommended as a reference. This score uses 90%, 85%, and 80% (the number of drops in oxygen saturation ≥3 times) as the standard for determining mild, moderate, and severe desaturation of oxygen, respectively. This is consistent with the diagnostic criteria in the 2007 edition of the “Draft Guidelines for the Diagnosis and Treatment of Obstructive Sleep Apnea Hypopnea Syndrome in Children (Urumqi)” (8). The results of the Pearson analysis in this study showed that the OSA-18 score was positively correlated with OAHI, whereas LSaO_2_ and OAHI were negatively correlated; that is, the lower the LSaO_2_, the higher the OAHI, the more severe the disease, and the greater the impact on the quality of life of the children. This finding can be used as a reference index for OSA, and the area under the ROC curve was larger than that of the OSA-18 scale, indicating that LSaO_2_ has a better primary screening effect than the OSA-18 scale for children with OSA. Although intermittent hypoxia is one of the main pathophysiological mechanisms of OSA, not all apnea cases are accompanied by a decrease in oxygen saturation. Studies have found that LSaO_2_ has the highest sensitivity and specificity for identifying moderate-to-severe OSA, 82.33% and 92.31%, respectively (9). Therefore, LSaO_2_ alone cannot be used as a screening index for OSA. Wu (10) and others found that the detection thresholds of mild, moderate, and severe OSA using LSaO2 were 86%, 75%, and 83%, respectively, but the results of this study suggest that the optimal threshold for mild and moderate-to-severe OSA is 90%, which is quite different from the MOS score, which may be related to the small sample size and limited data.

The guideline points out that neither the LSaO2 nor the OSA-18 scale alone can be used as screening indicators for OSA, but the ROC curve of the diagnostic test using the combination of LSaO2 and the OSA-18 scale showed that the area under the ROC curve of LSaO2 was 0.669, that of the OSA-18 scale was 0.623, while the area under the ROC curve of the combined test of the two was 0.739, which was larger than that of using the two indicators alone. The Table 4 show that, the decrease in sensitivity was lower than the increase in specificity, meaning that although there was a probability of missed diagnosis, the risk of misdiagnosis was significantly reduced. This helps to reduce unnecessary anxiety for parents and the possibility of subsequent invasive surgeries. For children who may have been missed in diagnosis, if the symptoms of sleep disorders are relatively obvious but the combined test shows negative, it can be recommended that they undergo polysomnography for an accurate diagnosis. For the joint test method, this study combined two indicators to carry out series and parallel tests. The results showed that the parallel test was better than the series test in terms of sensitivity. Although the specificity was lower than that of the series test, the sensitivity increased. Therefore, the parallel test can better screening OSA when the two are used in combination, one of the two is an abnormal, OSA can be diagnosed. Especially when the OSA-18 score is higher than 59, moderate OSA can be diagnosed, which can play a good role in identifying moderate to severe OSA. For mild OSA, clinical intervention can be performed after determining the cause, and drug treatment can be administered according to clinical experience. When necessary, sleep posture correction and weight loss can be provided for children with obesity; however, surgery is not the first choice for treatment. This is also the most important question encountered by parents in the clinical work of doctors regarding the removal of tonsils/adenoids. According to the results of this study, if the OSA-18 scale and LSaO_2_ are used in combination, primary screening examinations for moderate and severe OSA can also be performed in primary hospitals without PSG. LSaO2 can be used to measure at night by blood oxygen monitor, and combined with OSA-18 scale, so doctor can answer the parents' questions.

Several limitations should be acknowledged. First, the sample size was relatively small, which may have influenced the stability of the estimated cut-off values, particularly for LSaO₂, and may partly explain differences from previously reported thresholds. Second, our cohort was recruited from a symptomatic clinical population (children with snoring, mouth breathing, and/or suspected apnea) and did not include asymptomatic healthy controls; consequently, there were relatively few participants with normal OAHI. This also resulted in a marked imbalance between the simple snoring group and the OSA group, which may affect the robustness of between-group comparisons and the stability of ROC-derived estimates; therefore, these findings should be interpreted with caution. Future studies with larger and more balanced cohorts—including healthy children and simple snorers—and, if feasible, multi-center enrollment are warranted to further validate the screening performance and refine thresholds. Third, the OSA-18 questionnaire is completed by parents and is inherently subject to subjective perception and potential information bias, which may vary with caregivers' educational and cultural backgrounds; standardized guidance during completion may help reduce such bias. Finally, although both OSA-18 and LSaO₂ showed statistically significant correlations with OAHI, the correlation coefficients were modest, which may also be related to sample size and the inherent differences between questionnaire-based assessment, oximetry, and PSG-derived indices.

## 5 Conclusion

Polysomnography (PSG) remains the diagnostic gold standard for pediatric obstructive sleep apnea (OSA); however, its limited availability in many primary hospitals creates a substantial gap between clinical demand and diagnostic capacity. In this retrospective study of children with sleep-disordered breathing, we found that combining the OSA-18 questionnaire with the lowest nocturnal oxygen saturation (LSaO₂) provided better discriminatory performance for identifying clinically meaningful OSA—particularly moderate-to-severe disease—than either measure alone. When applied as a parallel testing strategy, this combined approach achieved high sensitivity, supporting its role as a practical preliminary screening and triage tool in resource-limited settings. Importantly, this method is not intended to replace PSG; rather, it may help frontline clinicians prioritize referrals and guide early clinical decision-making for symptomatic children when PSG is not readily accessible. Further multicenter studies with larger sample sizes and inclusion of healthy controls are warranted to validate the proposed thresholds and refine performance across the full spectrum of disease severity.

## Data Availability

The original contributions presented in the study are included in the article/Supplementary Material, further inquiries can be directed to the corresponding author.
